# Effect of radiant exposure and UV accelerated aging on physico-chemical and mechanical properties of composite resins

**DOI:** 10.1590/1678-7757-2018-0075

**Published:** 2019-01-07

**Authors:** Carlos Enrique Cuevas-Suárez, Carine Tais Walter Meereis, Norma D’accorso, Ricardo Macchi, Adriana Leticia Ancona-Meza, Eliezer Zamarripa-Calderón

**Affiliations:** 1 Universidad Autónoma del Estado de Hidalgo, Instituto de Ciencias de la Salud, Área Académica de Odontología, Pachuca de Soto, México; 2 Universidade Federal dos Vales do Jequitinhonha e Mucuri, Programa de Pós-Graduação em Odontologia, Diamantina, Minas Gerais, Brasil; 3 Universidad de Buenos Aires, Facultad de Ciencias Exactas y Naturales, Departamento de Química Orgánica, Buenos Aires, Argentina; 4 Universidad de Buenos Aires, Facultad de Odontología, Departamento de Materiales Dentales, Buenos Aires, Argentina

**Keywords:** Composite resins, Aging, Polymerization

## Abstract

Currently, there is no consensus in terms of defining the minimum radiant exposure values necessary for achieving adequate properties of composite resin. In addition, the long-term influence that radiant exposure has on the properties of composite resins is still questionable. Objective: The objective of this study was to evaluate the effect of radiant exposure and UV accelerated aging on the physico-chemical and mechanical properties of micro-hybrid and nanofilled composite resins. Material and Methods: A nanofilled (Filtek Supreme; 3M ESPE) and a micro-hybrid composite resin (Filtek Z250; 3M ESPE) were investigated under different radiant exposures (3.75, 9, and 24 J/cm^2^) and UV accelerated aging protocols (0, 500, 1000, and 1500 aging hours). The degree of conversion (DC), flexural strength (FS), modulus (M), water sorption (WS), and solubility (WL) were evaluated. The results obtained were analyzed using two-way ANOVA and Tukey's test. Comparisons were performed using a significance level of α=0.05. Results: The DC, FS, and M were found to be significantly influenced by both radiant exposure and accelerated aging time. The DC and EM increased with radiant exposure in the no-aging group (0-hour aging) for both micro-hybrid and nanofilled composites, whereas no correlation was found after accelerated aging protocols. WS and WL of micro-hybrid and nanofilled composite resins were scarcely affected by radiant exposure (p>0.05), whereas they were significantly reduced by accelerated aging (p<0.001). Conclusions: Although increasing radiant exposure affected the degree of conversion and mechanical properties of micro-hybrid and nanofilled composites, no influence on the hydrolytic degradation of the material was observed. In contrast, UV accelerated aging affected both the physico-chemical and mechanical properties of the composites.

## Introduction

Dental resin-based composites comprise two major components: an organic matrix composed of monomers, an initiation system, accelerators, and inhibitors; and inorganic filler particles, as well as a silane derivative as coupling agents.^1^ Both organic and inorganic phases have an influence on the chemical, physical and mechanical properties of the composite. The coupling agent used, and the characteristics of the filler (concentration, type, size, and distribution) can affect the overall properties of composites resins.^2^ Additionally, the degree of conversion of the organic matrix is another determinant of several properties of restorative resins, being signiﬁcantly correlated to many material characteristics, such as mechanical properties, polymerization shrinkage, wear resistance, and monomer elution.^3^ Considering this, any factor that affects the degree of conversion from composite resins can significantly influence their physico-chemical and mechanical properties.

Previous studies have established a direct relationship between the degree of conversion and the radiant exposure (J/cm^2^) emitted by the light source.^4^ ^,^ ^5^ On this basis, a common tendency among clinicians is to increase irradiance (mW/cm^2^) and/or exposure time (seconds) to use high radiant exposure levels during light curing.^3^ However, this relation is not linear and, consequently, no significant increase in the degree of conversion is expected above certain radiant exposure values.^6^ Moreover, the use of high radiant exposure has been associated with the development of higher polymerization shrinkage stress levels.^7^

Levels of radiant exposure are highly dependent on the material being investigated, like constitution, thickness, shade and refraction index.^8^ Currently, dental literature shows controversy in terms of defining the minimum radiant exposure values necessary for achieving adequate mechanical properties of photopolymerizable resin-based materials. Thus, levels of 4–6 J/cm^2^ are considered sufficient in some reports,^9^ whereas other studies^10^ warn about the impairment risk of the composite strength when using radiant exposures below 12 or 16 J/cm^2^. Furthermore, the long-term influence of radiant exposure on the physico-chemical and mechanical properties of composite resins is still questionable.

For the study of the behavior and longevity of dental restorative materials, accelerated aging under standardized laboratory conditions is an alternative because it allows the simulation of clinical long-term conditions in a short time period. Methods like water or water-ethanol immersion, artificial saliva immersion, hydrothermal cycling and ultraviolet (UV) accelerated aging system have been widely used for this purpose.^11^ In this study, we aimed to evaluate the effect of radiant exposure and accelerated aging on the physico-chemical and mechanical properties of micro-hybrid and nanofilled composite resins. The null hypothesis tested was that radiant exposure and UV accelerated aging affect neither the chemical nor the mechanical properties of micro-hybrid and nanofilled composite resins.

## Material and methods

### Study design

**Figure 1:**
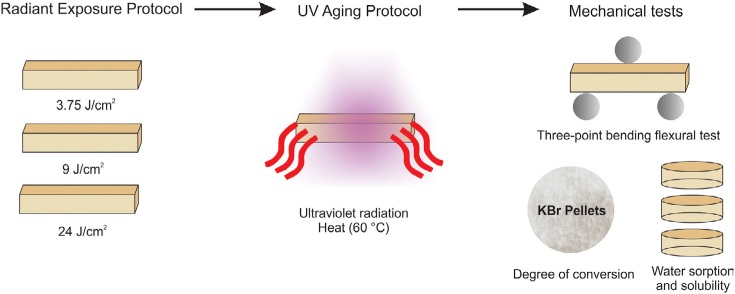
Schematic representation of the study design

**Figure 2:**
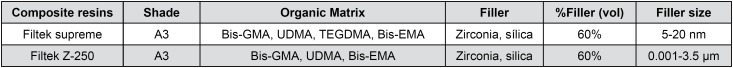
Constitution of the composite resins used in this study

In this study, chemical, mechanical and morphological characterizations were performed according to these factors: (1) photopolymerizable composite resins at two levels: a nanofilled (Filtek Supreme; 3M ESPE, St. Paul, MN, USA) and micro-hybrid composite resin (Filtek Z250; 3M ESPE, St. Paul, MN, USA); (2) radiant exposure at three levels: 3.75 J/cm^2^, 9 J/cm^2^ and and 24 J/cm^2^; and (3) UV accelerated aging protocol at four levels: 0, 500, 1000 and 1500 aging hours (Figure 1). The constitution of the composite resins evaluated in this study is described in the Figure 2.

### Curing protocols

Composite resins were photopolymerized using halogen light-polymerization unit (Spectrum 800; Dentsply Caulk, Milford, MA, USA). The specimens were subjected to three radiant exposure protocols: 3.75 J/cm^2^ (250 mW/cm^2^ for 15 s), 9 J/cm^2^ (450 mW/cm^2^ for 20 s), and 24 J/cm^2^ (800 mW/cm^2^ for 30 s). The intensity of light irradiation was periodically monitored using a digital radiometer (Cure Rite #800; EFOS Incorporation, Williamsville, NY, USA).

### Accelerated aging protocol

The specimens of composite resin were exposed to UV accelerated aging for 500, 1000, and 1500 hours, which represent approximately 1, 2, and 3 years of clinical use, respectively.^12^ The specimens were placed in a QUV/Basic weathering chamber (Q-Panel Lab. Cleveland, OH, USA) equipped with UVB 313 fluorescent tubes emitting a maximum peak of 313 nm under a relative humidity of 100%. Then, a course of 4 hours of UV radiation at 60°C and 4 hours of vapor condensation at 40°C was applied.

### Degree of conversion

The degree of conversion was measured using a FTIR spectrometer (Magna 590; Nicolete Instrument Corporation, Madison, WI, USA.). First, 100 mg of each uncured composite resin was dissolved in 5 mL of dimethyl sulfoxide. An aliquot of this solution was then placed on KBr pellets and the corresponding FTIR spectrum was recorded. For cured composite resins, disk specimens were prepared by filling the uncured composite resin into stainless steel mold (6 mm x 2 mm). The samples were light cured according to the curing protocol described above, being subsequently subjected to the accelerated aging protocol. Once the UV accelerated aging was concluded, each sample was ground with mortar and pestle, and 100 mg of cured composite was coated on KBr pellets to obtain a thin film. FTIR spectra of each sample (n=5) were recorded. To quantify the concentration of double bonds, the heights of the aliphatic and aromatic uC=C absorption bands, which appeared at 1638 and 1609 cm^−1^, respectively, were determined. The aromatic stretching C=C vibration was used as internal standard because its absorption band area remained constant after the polymerization process. The degree of conversion of the specimens was determined according to the formula:

$$\text{Degree of conversion}\left(\%\right)=100\left[1–\left(A_{1638}/A_{1609}\right)\text{polymer}/\left(A_{1638}/A_{1609}\right)\text{monomer}\right]$$

### Flexural properties

Flexural strength (FS) was evaluated in accordance with International Standard Organization Specification No. 4049,^13^ whereas the modulus (M) was evaluated using the square section of the flexural mechanical properties. Bar specimens (25 mm × 2 mm × 2 mm; n=10) were prepared inserting the uncured samples into a stainless-steel mold. The samples were irradiated on both sides using the curing protocol described above. The specimen dimensions were measured using a digital caliper (Mod. CD-6”C; Mitutoyo, Tokyo, Japan). After removing the specimens of the mold, they were subjected to the UV accelerated aging process described above. Once the UV accelerated aging process was concluded, flexural properties were evaluated by performing a three-point bending test using a universal mechanical testing machine (Instron 1100; Norwood, MA, USA). The mechanical test was performed at a cross-head speed of 1.00 mm/minute until fracture of the specimen. The values of FS (MPa) and M(GPa) were calculated from the load-displacement curve using the following formulas:

$$\begin{array}{l} \text{FS}=3\text{FI/}2{\text{bh}}^{2}\\ M=F_{1}I^{3}/4{\text{bh}}^{3}d, \end{array}$$

where F1 is the load, in newtons (N), exerted on the specimen; d is the deflection in millimeters (mm), corresponding to the load F1; F is the maximum load (N) exerted on the specimen at the point fracture, l the distance (mm) between supports, h the height (mm) of the specimen, and b the width (mm) of the specimen.

### Water sorption and solubility

The water sorption (WS) and solubility (WL) were evaluated according to ISO 4049.^13^ For such, composite disks (n=10) were prepared (15 mm in diameter and 1 mm in thickness), and the samples were polymerized following the curing protocol described above and subjected to the different UV accelerated aging processes.

Once the UV accelerated aging protocol was concluded, the samples were stored inside a desiccator and their masses were monitored daily until a constant value *m_1_* was attained, which was considered when the variation of two weights was less than 0.1 mg. After that, the diameter and thickness of the specimens were measured to obtain the volume of each one (V). Then, these specimens were immersed in distilled water at 37°C for 7 days and, after this time, the specimens were air-dried for 15 s and weighted to obtain the *m_2_* mass. Finally, the samples were stored again in a desiccator and were monitored until a constant mass was acquired (*m_3_*). The values of W_sp_ and W_sl_ were calculated using the following equations:

$$\begin{array}{l} W_{\text{sp}}=\left(m_{2}–m_{3}\right)/V\\ W_{\text{sl}}=\left(m_{1}–m_{3}\right)/V, \end{array}$$

### Environmental scanning electron microscopy

Three cylindrical specimens (15×1 mm) for each group were fabricated and exposed to UV accelerated aging process according to section 2.3. After aging processes, its surface morphology was analyzed using an environmental scanning electron microscope (ESEM Philips Electro Scan Mod. 2010; Philips, Andover, MA, USA) operated at 10kV and a pressure of 3 Torr. Different images at 1500× magnifications were obtained.

### Statistical analysis

The data obtained for each composite resin type was individually analyzed to check normality and homoscedasticity. Two-way ANOVA was conducted to evaluate the effect of radiant exposure and accelerate aging time on dependent variables. *Post hoc* multiple comparisons were performed using Tukey's test. Additional analysis using correlation between dependent variables and radiant exposure or accelerated aging time was performed through Pearson's product-moment correlation and linear regression analysis. *R* values over 0.70 were considered to correspond to a strong relation. A significance level of α=0.05 was used for all analyses. The statistical analyses were performed using IBM SPSS Statistics 20 Software (Armonk, NY, USA).

## Results

**Figure 3:**
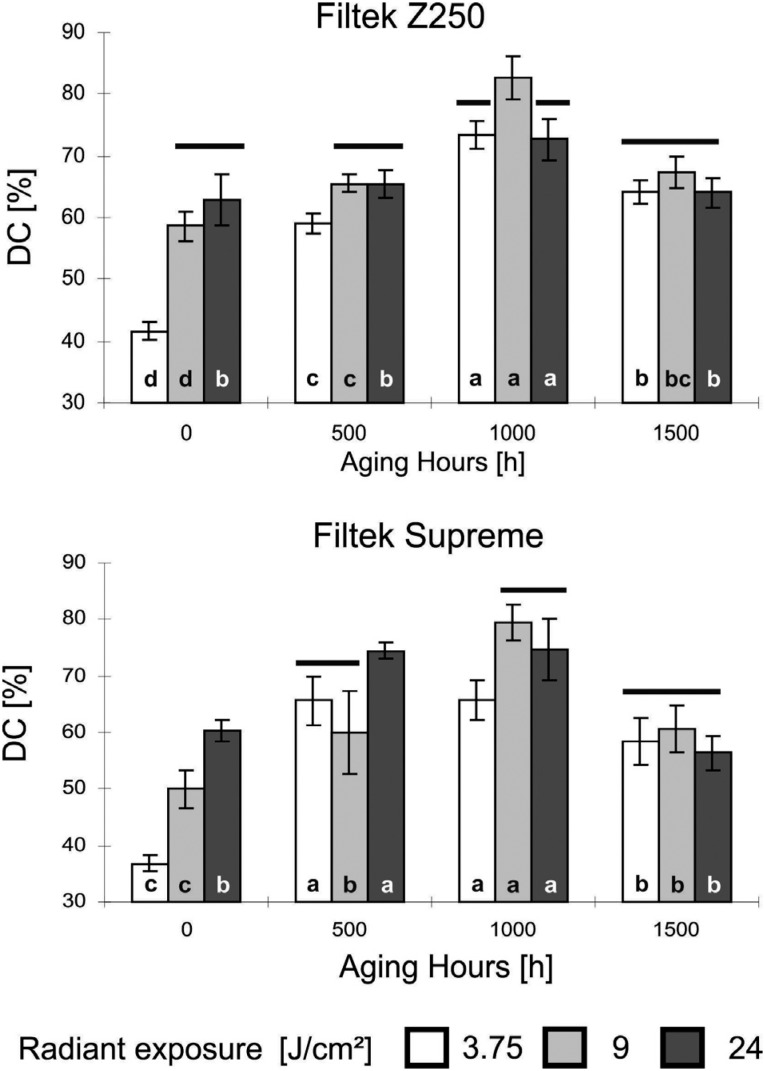
Degree of conversion for Filtek Z250 (top) and Filtek Supreme (bottom) at different radiant exposures and UV accelerated aging hours. Columns under the same horizontal line indicate no differences between radiant exposure for each period of aging hours. Different lowercase letters indicate differences between aging hours within each radiant exposure

The degree of conversion from the composite resins was found to be significantly influenced by both radiant exposure (p<0.001) and accelerated aging time (p<0.001), and an interaction between these two variables was observed (p<0.001) (Figure 3). Thus, the degree of conversion increased with radiant exposure in the no-aging group (0-hour aging group) for both micro-hybrid (r=0.79; p<0.001) and nanofilled (r=0.91; p<0.001) composites, but no correlation was found after aging for 500, 1000, and 1500 hours. Regardless of the radiant exposure, the highest degree of conversion was achieved after aging for 1000 hours and the lowest was obtained with the 0-hour aging group for both micro-hybrid and nanofilled composites.

**Figure 4:**
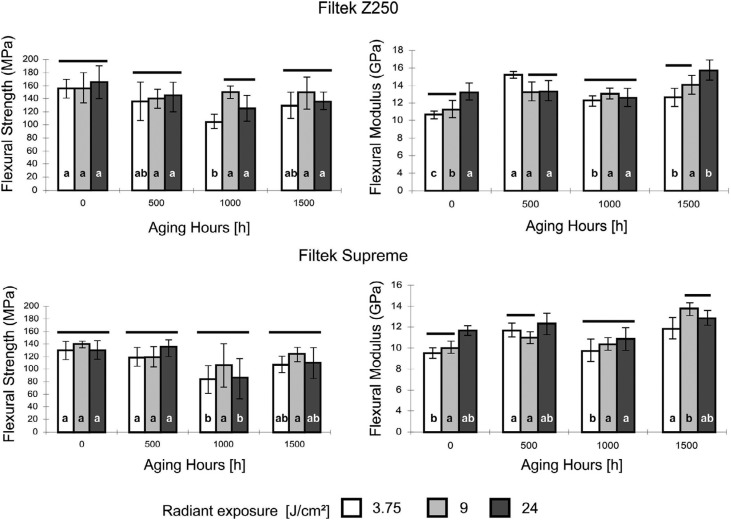
Flexural Strength and Flexural Modulus for Filtek Z250 (top) and Filtek Supreme (bottom) at different radiant exposures and UV accelerated aging hours. Columns under the same horizontal line indicate no differences between radiant exposure for each period of aging hours. Different lowercase letters indicate differences between aging hours within each radiant exposure

Although both the FS and modulus of the composite resins were significantly affected by radiant exposure and aging time, the interaction between these two factors was only significant for the modulus in both composites (p<0.001) (Figure 4). An increase in the modulus with radiant exposure was observed in the 0-hour aging group for both micro-hybrid (r=0.83; p<0.001) and nanofilled composite (r=0.88; p<0.001), but no correlation was found after 500, 1000, and 1500 aging hours.

**Figure 5:**
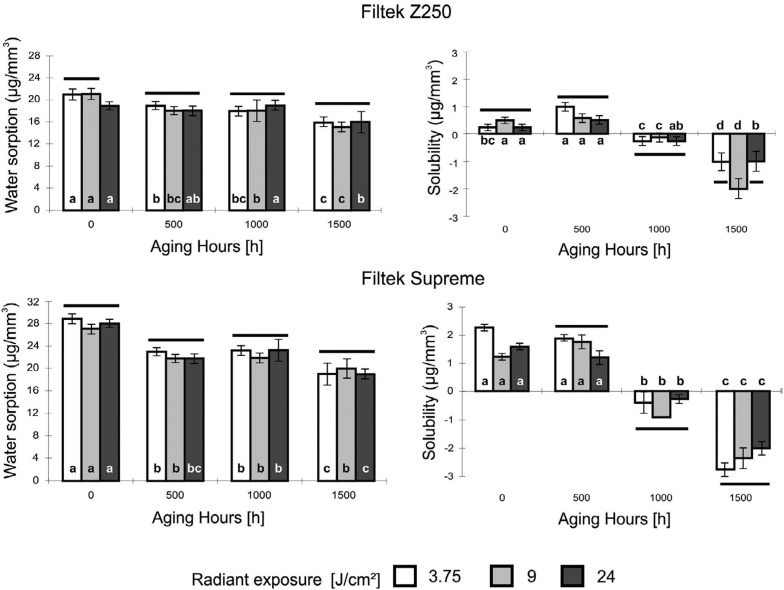
Water sorption and solubility for Filtek Z250 (top) and Filtek Supreme (bottom) at different radiant exposures and UV accelerated aging hours. Columns under the same horizontal line indicate no differences between radiant exposure for each period of aging hours. Different lowercase letters indicate differences between aging hours within each radiant exposure

Water sorption and solubility of micro-hybrid and nanofilled composite resins were scarcely affected by radiant exposure (p>0.05). In contrast, these parameters were significantly reduced when the UV accelerated aging time increased in 3.75, 9, and 24 J/cm^2^ radiant exposures for both micro-hybrid (r≥0.80; p<0.001) and nanofilled (r≥0.60; p≤0.02) composites (Figure 5).

**Figure 6:**
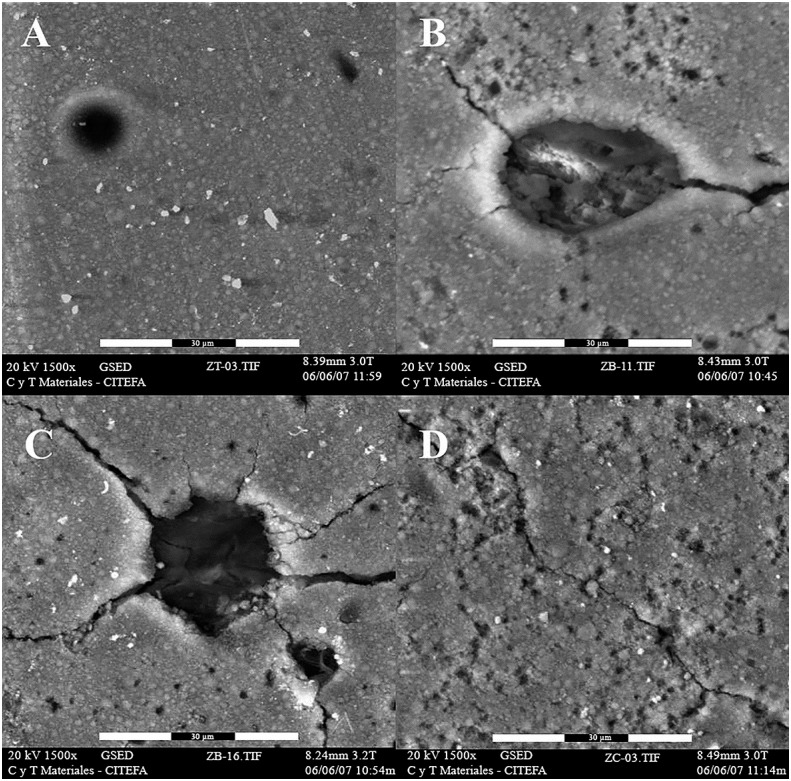
Representative ESEM micrographs of Filtek Z250 at 0 (A), 500 (B), 1000 (C) and 1500 (D) UV accelerated aging hours. Images were taken at 1500x magnification

**Figure 7:**
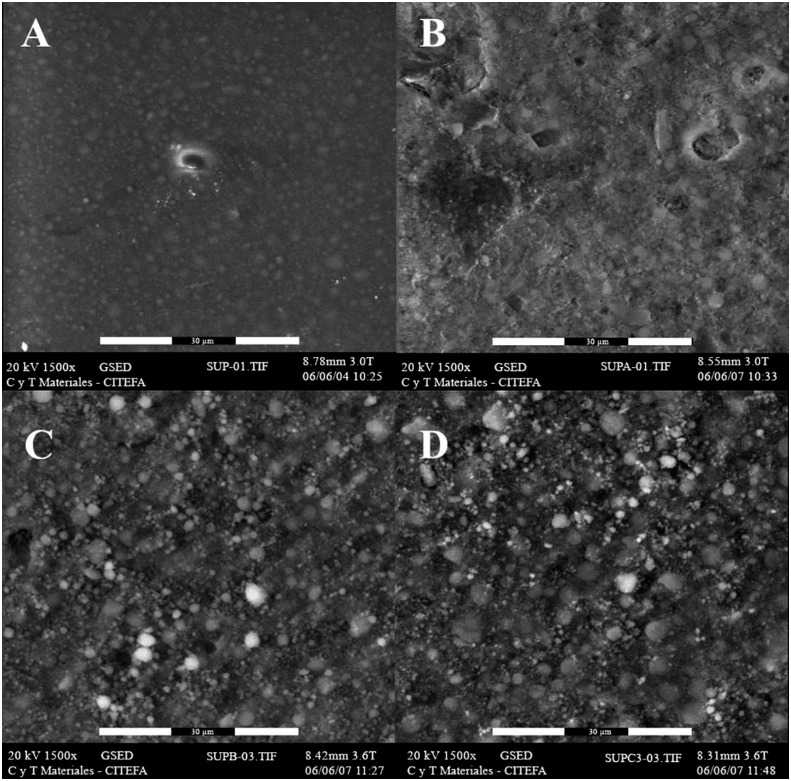
Representative ESEM micrographs of Filtek Supreme at 0 (A), 500 (B), 1000 (C) and 1500 (D) UV accelerated aging hours. Images were taken at 1500x magnification

Figures 6 and 7 show the SEM micrographs obtained for the micro-hybrid and nanofilled composites, respectively, after being subjected to UV accelerated aging. The micro-hybrid composite Filtek Z250 revealed the presence of cracks and pores after aging for 500 hours (Figure 6B). This becomes more evident after aging for 1000 (Figure 6C) and 1500 hours (Figure 6D). The nanocomposite Filtek Supreme presented a surface with apparently increased roughness after aging for 500 hours (Figure 7B). The degradation of the organic matrix is evident after aging of 1000 and 1500 hours, since the filler particles seem to be more exposed (Figure 7C-D).

## Discussion

An improvement in the physico-chemical and mechanical properties of resin-based materials has been reported to take place upon increased radiant exposure.^3^ ^-^ ^5^ However, the findings of this study indicate that the degree of conversion and the mechanical properties of micro-hybrid and nanofilled composite resins depend on both radiant exposure and UV accelerated aging time, whereas the hydrolytic degradation of the material depends only on the latter. Thus, the null hypothesis was rejected.

The degree of conversion increased with radiant exposure in the 0-hour aging group for both micro-hybrid and nanofilled composites, but no correlation was found after aging for 500, 1000, and 1500 hours. These results corroborate with other studies that have also demonstrated an initial direct relation between radiant exposure and double-bond conversion.^14^ ^,^ ^15^ Polymerization kinetics theory of light-activated composite resins suggest that the production of free radicals increases with radiant exposure, which is accompanied by an increment in the formation of multiple growth centers and a subsequent increase in the tendency to form a high cross-linked polymer.^16^ Also, when materials are subjected to high levels of irradiance, the resulting rise in exothermic heat within the polymeric matrix could provide polymer chains with greater mobility, which would account for a higher degree of conversion observed in composites irradiated with 24 J/cm^2^. Achieving high values of degree of conversion is not only important to improve the mechanical properties of the material, but also has an important effect on the biological response of the pulp-dentinal complex, since unreacted monomers could be released into the medium and may irritate the pulp, generating an inflammatory response.^17^

Despite the initial positive effect, it is worth mentioning that the influence of radiant exposure on the degree of conversion was found to disappear after 500, 1000 and 1500 hours of accelerated aging. This behavior could be attributed to the effects of post-cure polymerization, heat, and UV irradiation. It is well known that the polymerization reaction of light-activated composites continues even after the exposure to visible light irradiation is interrupted.^18^ Although, during initial polymerization, free radicals located at the functional groups of unreacted molecules can be quenched within the polymer network due to rapid increase in viscosity,^19^ our findings suggest that even low radiant exposures to visible light can provide the unreacted molecules with sufficient molecular mobility that allows for additional polymerization in the organic matrix to proceed.

The maximum values of conversion were achieved after aging for 1000 hours, which is likely due to the effect of heat and UV irradiation on the methacrylate groups. Heat and UV radiation are commonly used to initiate the polymerization of acrylate monomers.^20^ Accordingly, photoinitiators that absorb light at the ultraviolet spectral wavelength region can be added to resin-based dental composites.^21^ When a monomer is exposed to UV irradiation in the presence of initiators, large amounts of free radicals are immediately generated and strongly cross-linked polymer networks can be formed.^22^ On the other hand, the increase in the internal temperature of the material allows for molecular relaxation and, as a consequence, an increase in the polymer chain mobility.^23^ In such a condition, trapped free radicals could react with unpolymerized monomers, favoring additional cross-linking.^24^ We observed a decrease in the degree of conversion after aging for 1500 hours, which could be attributed to some kind of degradation of the organic matrix. This degradation could be caused by the production of methacrylic acid by scissoring residual monomer or by the unzipping of polymer chains from BisGMA/TEGDMA copolymers.^25^

The degree of conversion of a material is significantly correlated to other important material characteristics, such as mechanical properties, volumetric shrinkage, and monomer elution.^26^ According to a recent review, the flexural strength of resin-based composites may be used as a predictor of clinical wear.^27^ In this study, the FS and FM of composite resins were found to be significantly affected by radiant exposure and accelerated aging time. When examining the FS, no interaction was found between radiant exposure and aging time, whereas such an interaction was observed in the case of FM. Increasing radiant exposure of curing composites has been shown to enhance the crosslinking density of polymers and to increase the FM of dental composites.^28^ Additionally, an increased polymerization shrinkage stress of the dental composite resins irradiated with higher radiant exposure could be expected due to their increased FM values.^29^ Flexural strength was shown to be less sensitive to variations in degree of conversion than flexural modulus,^30^ which could explain the lack of influence of low radiant exposures on the former property. These results corroborate with other studies that have also demonstrated a direct relation between radiant exposure and flexural properties of composite resins.^31^ ^,^ ^32^

According to our results, UV accelerated aging had a significant effect on water sorption and solubility. In contrast, these properties were not affected by radiant exposure. Water sorption and solubility values decreased when accelerated aging time is increased under 3.75, 9, and 24 J/cm^2^ radiant exposures for both micro-hybrid and nanofilled composites. This behavior could be correlated with the degree of conversion and crosslinking density of the polymers formed after aging for 500, 1000, and 1500 hours. High crosslinking density has been detected in polymeric materials with higher degree of conversion, where a reduction in the solvent uptake and swelling as a result of the reduced free volume in the network is observed.^33^

The surface degradation in all samples was evident after the first 500 hours of aging, likely due to the incidence of UV radiation and the presence of water in the environment causing a physical alteration of the composite surface that led to chemical degradation. These surface modifications did not lead to significant variations in chemical and mechanical properties of the materials evaluated in this study, however, the presence of cracks, voids, and irregularities in the surface of all samples could promote the adherence and colonization of microorganisms.^34^ Also, the increase of the surface roughness is not considered acceptable clinically.^35^

Filler characteristics are a critical factor in the determination of the properties of composites. Nanofilled composites show low light transmittance,^36^ so light that passes through the composite resin is scattered and reduced. This phenomenon could be expected to cause a decrease in the rate of free radicals available for polymerization, which would therefore affect the physico-chemical and mechanical properties of the composites. However, the nanofilled and micro-hybrid composite resins investigated here exhibited similar sensitivity to variations in physico-chemical and mechanical properties after radiant exposure and accelerated aging.

Based on our findings, the polymerization of the micro-hybrid and nanofilled composite resins using a radiant exposure of at least 9 J/cm^2^ seems to be enough to obtain adequate initial and long-term properties. Since this radiant exposure was obtained using relatively low light irradiation (450 mW/cm^2^), a reduction in the polymerization stress is expected, limiting the detrimental effects that this property represents. Additionally, this radiant exposure could be obtained using relatively low exposition time (20 s), leaving it as a clinically viable photopolymerization protocol.

## Conclusions

We have demonstrated that an increase in radiant exposure affects the degree of conversion and mechanical properties of micro-hybrid and nanofilled composite resins, whereas not affecting the hydrolytic degradation of the material. In contrast, UV accelerated aging affects both the physico-chemical and mechanical properties of the composites.
